# Mucinous adenocarcinoma emerging in sigmoid colon neovagina 40 years after its creation: a case report

**DOI:** 10.1186/s12957-015-0636-0

**Published:** 2015-07-11

**Authors:** Yoshiaki Kita, Shinichiro Mori, Kenji Baba, Yasuto Uchikado, Takaaki Arigami, Toshihiko Idesako, Hiroshi Okumura, Sumiya Ishigami, Masayuki Nakagawa, Shoji Natsugoe

**Affiliations:** Department of Digestive Surgery, Breast and Thyroid Surgery, Graduate School of Medicine, Kagoshima University, Sakuragaoka 8-35-1, Kagoshima, 890-8520 Japan; Department of Urology, Graduate School of Medicine, Kagoshima University, Sakuragaoka 8-35-1, Kagoshima, 890-8520 Japan

**Keywords:** Colon cancer, Neovagina, Surgery

## Abstract

**Background:**

We reported our experience of adenocarcinoma of sigmoid colon neovagina.

**Case presentation:**

A 67-year-old female with a history of neovagina construction for Rokitansky syndrome complained of vaginal bleeding. She had a mucinous adenocarcinoma at the anterior aspect of the neovagina. Her original surgery, using sigmoid colon to construct the artificial vagina, was 40 years ago

**Conclusions:**

This patient’s case may contribute to our understanding of carcinogenesis in the colon.

## Background

Diverse operative methods have been employed for the treatment of vaginal agenesis with attendance advantages and disadvantages. Skin, peritoneum, small intestine, and colon have all been used to create neovaginas, but no method has a major consensus due to the relative rarity of this condition [1]. Considering the incidence of vaginal agenesis itself, malignancy arising in the neovagina is extremely rare.

Here, we report a patient with adenocarcinoma of the neovagina; the substitute for vagina was related 40 years prior from segment of sigmoid colon. We describe “tips and traps” of surgery for malignancy in an artificial vagina, and we attempt an etiological understanding of this type of colon cancer by reviewing previous reports.

## Case presentation

Forty years ago, a 27-year-old Japanese woman came to the outpatient clinic of Kagoshima University Hospital with primary amenorrhea. Examination revealed an absent vagina and a rudimentary uterus, and she was diagnosed with Mayer-Rokitansky-Küster-Hauser syndrome. She promptly underwent surgery to construct a neovagina, using a segment of sigmoid colon. She married 2 years after surgery and was able to successfully have sexual intercourse for about 20 years.

Forty years after surgery, at the age of 67, she came to our outpatient clinic with the chief complaint of bleeding and a hard nodule. Endoscopy inside neovagina disclosed a protruding mass at the anterior wall of the vagina, immediately inside the introitus, involving the urethral meatus. A biopsy revealed poorly differentiated adenocarcinoma, and both vaginal and urine cytology were positive for cancer cells. CT, MRI, ultrasonography (US), and PET were performed; there was no evidence of lymphadenopathy or distant metastasis. Colonoscopy did not reveal any abnormalities, and her CA19-9 level was normal (17.1 U/ml), CEA level was increased slightly to 6.8 U/ml. We diagnosed colon cancer originating in the artificial vagina, encompassing the urethral meatus but without lymph node involvement or distant metastasis. Moreover, her family history excluded hereditary disease. Our plan was for curative resection.

**Fig. 1:**
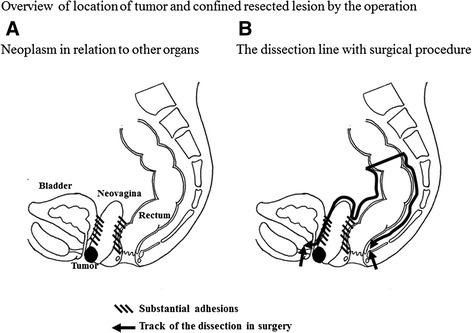
Overview of neoplasm location. **a** Neoplasm in relation to other organs. **b** The dissection line with surgical procedure

**Fig. 2:**
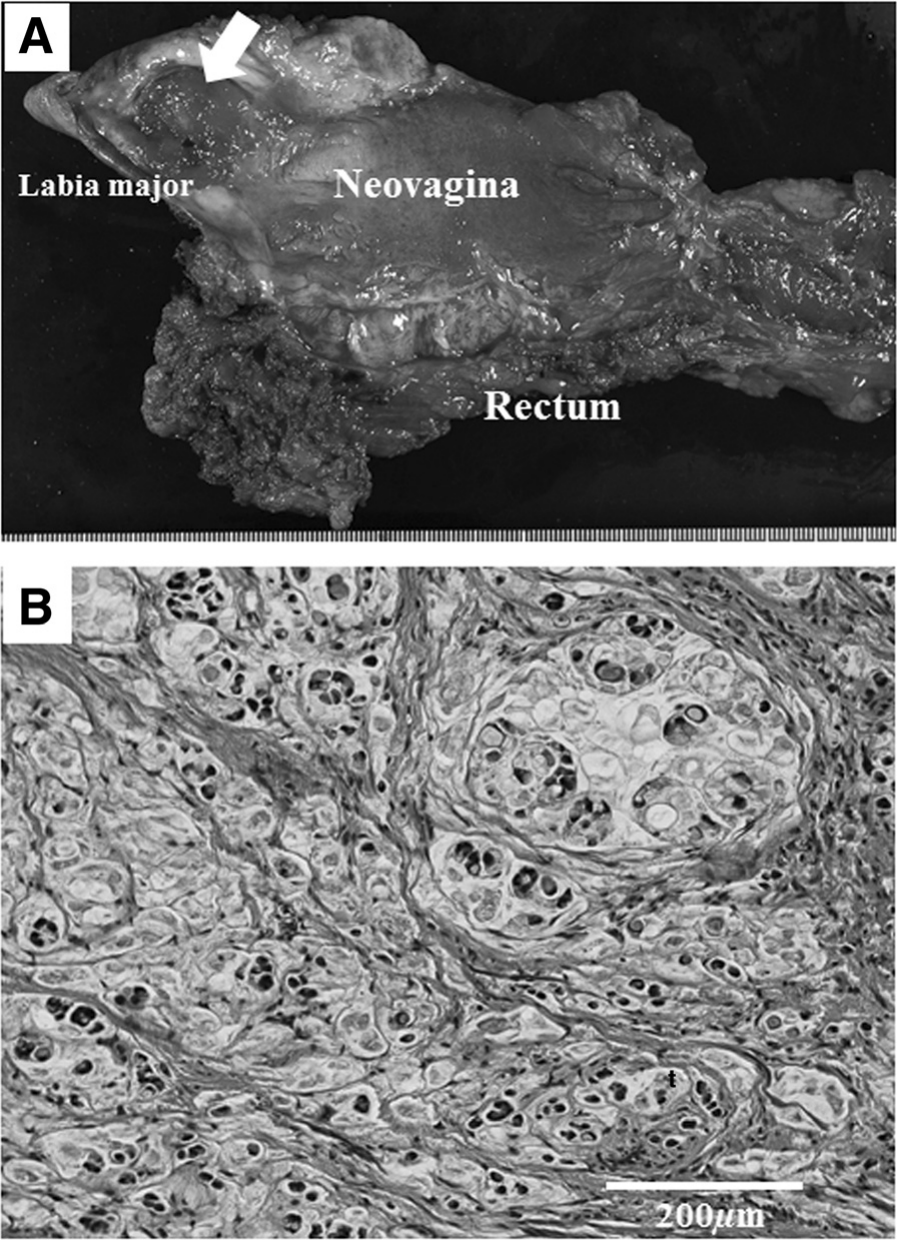
**a** Macroscopic sagittal section. Type 1 colon cancer, 2.5 × 2.5 cm (*arrow*). The specimen includes the ureteral orifice. The resected neovagina has thick walls. **b** Microscopic finding (hematoxylin and eosin staining; magnification; ×200). The neoplasm consists of mucinous carcinoma cells with signet-ring cells

Although we tried to resect only the neovagina, using manipulation from both the laparoscopic intraperitoneal approach and anal approaches, substantial adhesions necessitated removal of the sigmoid colon and rectum as well. We performed an abdominoperineal resection, including the urethral meatus, with resection of the neovagina (Fig. 1a, b). The resected neovagina had thick walls, and we were able to obtain an adequate surgical margin. Pathological examination confirmed that the margins of the resected rectum and urethral meatus were also clear (Fig. 2a). The final pathological diagnosis was mucinous adenocarcinoma of transplanted colon tissue, involving urethral meatus but without nodal metastasis (Fig. 2b). According to Japanese classification of colorectal cancer and the treatment guideline of colorectal cancer in the Japanese Society for Cancer of the Colon and Rectum, the patient was categorized as high-risk stage II colorectal cancer. Therefore, she underwent adjuvant therapies to took S-1 orally for half year and have been followed without recurrence of cancer.

### Discussion

Mayer-Rokitansky-Küster-Hauser syndrome is a disorder that presents as Mullerian agenesis, and affected woman may have abnormalities of the internal genitalia that include the absence of both the uterus and the upper two-thirds of the vagina [2]. Several methods of neovaginal construction have been established; either a split-thickness or full thickness skin graft may be used, and surgeons have also employed the gracilis myocutaneous flap, small intestine, and labia majora [1, 3, 4].

Moreover, the surgery by these methods has been tried under the laparoscopic surgery [5]. The use of sigmoid colon, 15 cm in diameter, was first reported by E. Ruge in 1914. The sigmoid colon has advantages over the small bowel, narrowing or stenosis is less likely. In addition, the sigmoid colon has large lumen and is therefore more satisfactory for intercourse. Finally, its thicker mucosa is less vulnerable to trauma induced by intercourse.

Primary vaginal carcinoma is extremely rare, according for reportedly 1–3 % of all gynecological malignancies [6, 7]. There are few reports of carcinoma arising in the neovagina. Hiroi et al. summarized 11 worldwide cases of neovaginal carcinoma and note that the pathological characteristics are likely to be associated with the tissue used for the reconstruction [8]. For instance, eight patients with skin graft neovaginas had squamous cell carcinoma, the whole others with intestinal neovaginas had adenocarcinoma. Two of the 11 reported malignancies were in sigmoid colon neovaginas: the first was reported in 1938, without any detailed information, and the second case was reported by Hiroi et al. This makes ours the third case worldwide.

Previous reports have not given any treatment details, including those of the surgical procedure used, for carcinoma arising in the neovagina. Reports describe only resection of the neovagina and adjuvant radiation [8]. Although we were initially intending to remove our patient’s neovagina, uterus, and uterine adnexae using laparoscopic techniques, substantial adhesions to the rectum and bladder did not allow for separation of this organ. Therefore, we had to employ abdominoperineal resection, including the urethral meatus, with resection of the neovagina. It is pivotal to take into account the possibility of significant adhesions when planning surgery for these patients.

The incidence of rectal and sigmoid colon cancer is relatively high. Diverse factors have been explored in relation to the genesis and development of colon cancer, including, lifestyle, environmental factors [9–11], and host factors [12, 13]. In our patient, we are confident that this cancer was sporadic colon cancer originally because endoscopic findings and this patient’s family history exclude hereditary non-polyposis colorectal cancer and familial adenomatous polyposis and mucinous adenocarcinoma arose in the transplanted sigmoid colon, which was unexposed to feces for 40 years. The overall incidence of mucinous carcinoma among all colorectal carcinomas ranges from 7.8 to 18 % [14] and is most frequently found in the right colon, followed by the rectum, and its incidence in sigmoid colon is relatively low [15]. Carcinogenesis is thought to be associated with inflammatory processes in the sigmoid colon including colitis, ulcerative colitis, and Crohn’s disease [15]. Chronic inflammation due to bacterial infection or a change in normal bacterial flora may be a possible explanation for the generation of mucinous carcinoma in ectopic sigmoid colon. Hiroi et al. have considered the possibility that frequent sexual intercourse may provoke malignancy through microinjury and subsequent inflammation [8]. Moreover, chemical stimulation from semen and urine may have a carcinogenic effect.

## Conclusions

We encountered a patient with a rare mucinous adenocarcinoma of the sigmoid colon neovagina. It is important to perform screening and surveillance endoscopy in both colon and neovagina. Moreover, we were able to complete curative surgery in the face of significant adhesions. Our report reinforces the hypothesis that chronic inflammation of the transplanted colon may play a pivotal rule in the oncogenic potential. We await the compilation of similar case reports.

### Consent

Written informed consent was obtained from the patient for publication of this case report and any accompany images. A copy of the written consent is available for review by the Editor of this journal.

## Abbreviations

CA19-9: cancer antigen 19-9
CEA: carcinoembryonic antigen
CT: computed tomography
MRI: magnetic resonance imaging
PET: positron emission tomography
US: ultrasonography
